# Effect of Huayu Tongluo Herbs on Reduction of Proteinuria via Inhibition of Wnt/*β*-Catenin Signaling Pathway in Diabetic Rats

**DOI:** 10.1155/2017/3054145

**Published:** 2017-06-01

**Authors:** Lu Bai, Beibei Huo, Zhiqiang Chen, Qian Guo, Jing Xu, Jing Fang, Jianghua Zhang, Fenfang Zhang

**Affiliations:** ^1^Hebei Medical University, Shijiazhuang, Hebei 050017, China; ^2^Hebei University of Chinese Medicine, Shijiazhuang, Hebei 050200, China; ^3^Affiliated Hospital of Hebei University of Traditional Chinese Medicine, Shijiazhuang, Hebei 050011, China

## Abstract

The study investigated the expression of Wnt/*β*-catenin pathway in diabetic rats and the intervention effect of Huayu Tongluo herbs (HTH). Ten rats were randomly selected as control group and the remaining rats were established as diabetic models. The diabetic rats were randomly divided into model group and HTH treatment group. The intervention was intragastric administration in all rats for 20 weeks. At the end of every 4 weeks, fasting blood glucose and 24 h urinary total protein quantitatively were measured. At the end of the 20th week, biochemical parameters and body weight were tested. The kidney tissues were observed under light microscope and transmission electron microscopy. We examined Wnt/beta-catenin signaling pathway key proteins and renal interstitial fibrosis related molecular markers expression. The results showed that HTH could reduce urinary protein excretion and relieve renal pathological damage. Wnt4, p-GSK3*β* (S9), and *β*-catenin expression were decreased in the signaling pathway, but GSK3*β* level was not changed by HTH in diabetic rats. Furthermore, the expressions of TGF-*β*_1_ and ILK were decreased, but the level of E-cadherin was increased in diabetic rats after treatment with HTH. This study demonstrated that HTH could inhibit the high expression of Wnt/*β*-catenin pathway in kidney of diabetic rats. The effect might be one of the main ways to reduce urinary protein excretion.

## 1. Introduction

In the past three decades, diabetes patients were obviously increased with the improvement of life conditions in China. A study estimating the world prevalence of diabetes among adults reported that diabetes affected 285 million adults in 2010 and would affect 439 million adults by 2030, which indicated a growing burden of diabetes worldwide [1]. So diabetes has emerged as a major global public health issue, and it can increase the risk of heart, brain, kidney, blood vessels, and nerve irreversible damage that would be a serious threat to human health and quality of life. Diabetic nephropathy (DN), one of the most serious and common chronic microvascular complications of diabetes mellitus (DM), is characterized by progressive proteinuria and glomerulosclerosis with renal interstitial fibrosis. It is one of the major causes of end-stage renal disease (ESRD), which brings heavy burden to society and family.

Proteinuria is the single most powerful risk factor for patients with diabetes nephropathy, and its mechanism is intricate and complex [2]. At present, the pathogenesis of proteinuria based on podocyte injury in DN patients has become a hot spot [3, 4]. Slit diaphragm molecules are located in the glomerular slit diaphragm, playing an important role in the development and function maintaining of the glomerular filtration barrier. Impairment of filtration barrier and loss of slit diaphragm protein are important causes of proteinuria. Studies reported that abnormal activation of Wnt/*β*-catenin signaling pathway mediating the podocyte injury in diabetic nephropathy caused a reduction in nephrin protein in the slit diaphragm, proliferation of mesangial cells, and renal interstitial fibrosis and proteinuria continued increasing. Thus, blocking the overexpression of this pathway can reduce podocyte injury [5–7].

From the perspective of Traditional Chinese Medicine (TCM), in addition to the pathogenesis of deficiency* Qi *and* Yin *of DM, DN has another primary pathogenesis, which is blood stasis blocking collaterals of kidney. And this has also been widely recognized in the field of TCM [8, 9]. Based on the pathogenesis of blood stasis blocking collaterals in DN, we demonstrated that Huayu Tongluo herbs (HTH) could reduce the loss of nephrin, podocin, CD2AP, and other proteins in podocyte slit diaphragm, protect podocytes, reduce renal fibrosis, and reduce urinary protein leakage [10–12]. According to our previous research, in this study, we established DN rat model to explore whether the kidney of the rat has abnormal expression of Wnt/*β*-catenin signaling pathway. Moreover, it is unclear whether the the fact that HTH could protect podocytes and reduce renal fibrosis is associated with Wnt/*β*-catenin pathway. Therefore, the goal of this research is to explore whether HTH could reduce proteinuria via inhibition of Wnt/*β*-catenin signaling pathway in diabetic rats.

## 2. Materials and Methods

### 2.1. Animals

A total of 40 adult (5-6 weeks) male Sprague-Dawley rats weighing about 100 g and 120 g were obtained from Experimental Animal Science Center of Hebei Medical University (SPF grade, license number SCXK (Ji) 2013-1-003). The rats were housed in a light-controlled (12 h/12 h light/dark cycle) room at 24 ± 1°C and humidity of 50%–70%. All rats had free access to food and water. The animals in the study were approved by the Ethics Committee of Hebei Medical University (number DW2013001), China. And our experiment was performed in accordance with Guidance Suggestions for the Care and Use of Laboratory Animals published by the Ministry of Science and Technology of the People's Republic of China.

### 2.2. Animal Modeling, Grouping, and Drugs

Forty male rats were randomly divided into normal control group (C group, *n* = 10) and Streptozotocin- (STZ, Enzo Life Science Co., Ltd. USA) injecting group (*n* = 30) by table of random number. The rats in control group were fed with common food (3.51 kcal/g), while other rats were fed with high-fat diet (4.43 kcal/g) according to the method described by us previously [10] for 6 consecutive weeks. Then rats in STZ-injecting group were intraperitoneally injected with STZ 35 mg/kg (diluted with 0.1 M pH 4.3 sodium citrate solution to 1%), and the injection must be finished within 30 min. Meanwhile, rats in C group were injected corresponding volume of sodium citrate solution. Seventy-two hours later, their tail vein blood was collected. When their level of blood glucose was higher than 16.7 mmoL/L, the diabetic rats model was considered to be successful. Three rats died within 72 h after being given STZ, and another rat was rejected. A completely randomization procedure was used in grouping. The successful modeling rats were randomly divided into model group (M group, *n* = 13) and HTH treatment group (HTH group, *n* = 13). Then the intervention was intragastric administration in all rats for 20 weeks. The rats of HTH group were treated with Huayu Tongluo herbal granule (4.59 g·kg^−1^·d^−1^, gavage) and the rats of C group and M group were gavaged with equal volume of drinking water. The dose was converted according to human usual dose, compatibility proportion, and the body surface area of rat to human [13]. During the study, no other intervention was given. Rats were sacrificed at 20th weekend after induction of diabetes. The rats were fasted overnight and were intraperitoneally anesthetized with 10% chloral hydrate (0.35 mL/100 g body weight). Tissue samples (blood, urine, and kidneys) were collected and stored in freezer at −80°C. Blood, urine, and renal tissue samples were measured and analyzed using a blinded test method.

HTH is composed of five Chinese Materia Medica (CMMs): Danshen (Radix Salviae Miltiorrhizae), Chuanxiong (Rhizoma Chuanxiong), Dilong (Pheretima Aspergillum), Shuizhi (Hirudo), and Quanxie (Scorpio) in a ratio of 27 : 26 : 10 : 30 : 20. All the CMMs were in the form of formula granules and were donated by Guangdong Yifang Pharmaceutical Co., Ltd. (Foshan, China) (Table 1).

### 2.3. Biochemical Parameters

Body weight was measured at 20th weekend and fasting blood glucose (FBG) was measured every 4 weeks by ONETOUGH Ultra rapid blood glucose meter and glucose test strips (Johnson & Johnson Medical Ltd., USA). Rats were kept in individual metabolic cages for 24 h urine collection at the end of 0, 4, 8, 12, 16, and 20 weeks of treatment, respectively, for the detection of urinary protein excretion. Blood samples were collected from abdominal aorta to test blood urea nitrogen (BUN) and serum creatinine (Scr) by a Hitachi automatic biochemical analyzer (7600-020, Hitachi, Tokyo, Japan).

### 2.4. Western Blot Assay

Total protein was extracted from renal cortex of rats and quantified by the BCA assay (Tiangen Biotech Co., Ltd, Beijing, China). Protein sample was diluted by 5x loading buffer and denatured at 100°C for 5 minutes. Equal amounts of protein were separated on 10% sodium dodecyl sulphate-polyacrylamide gel electrophoresis (SDS-PAGE) at 4°C. Following electrophoresis, the proteins were transferred to the polyvinylidene difluoride (PVDF) membranes (Millipore Corporation, Bedford, MA, USA). After blocking with 5% nonfat milk in TBST for 1 hour, the membranes were incubated with primary antibodies overnight at 4°C and then with secondary antibodies for 1 hour at 37°C. Optical density of the bands was scanned and quantified using the Odyssey infrared fluorescence imaging system (LI-COR, USA). The primary antibodies used were as follows: Wnt4 antibody (GTX101085, Gene Tex Inc., CA, USA, 1 : 500), GSK-3*β* and E-cadherin antibodies (WL01456, WL00941, Wanleibio Co., Ltd., Shenyang, China, 1 : 500), *β*-catenin and ILK antibodies (ab32572, ab52480, Abcam, Inc., UK, 1 : 1000), p-GSK3*β* (S9) and TGF-*β*_1_ antibodies (BS4084, BS1361, Bioworld Technology Inc., MN, USA, 1 : 1000), and *β*-actin antibody (D110001, Sangon Biotech Co., Ltd. Shanghai, China, 1 : 1000).

### 2.5. Quantitative Real-Time Polymerase Chain Reaction (qPCR) Assay

Total RNA of renal cortex was extracted using the TRIzol Reagent (Life Technologies Corporation, Carlsbad, CA, USA). Its concentrations and purity were determined by a Nanodrop 2000C spectrophotometer (Thermo Scientific, Wilmington, DE, USA). Then the RNA was reverse-transcribed into cDNA by FastQuant first-strand cDNA synthesis kit (Tiangen biotech Co., Ltd, Beijing, China). Gene-specific primers were designed by Primer 5.0 and synthesized by Sangon Biotech Co., Ltd. (Shanghai, China) (Table 2). Polymerase chain reactions were performed with Platinum® SYBR® Green qPCR SuperMix-UDG (Invitrogen Co., Carlsbad, CA, USA) on Eco Real-Time PCR System (Illumina, San Diego, CA, USA). Each reaction was carried out in triplicate. And the relative mRNA levels were calculated by the 2^−ΔΔCq^ method.

### 2.6. Immunohistochemistry Assay

Kidney tissues were cut off to make immunohistochemistry sections and then deparaffinized. The epitopes were retrieved by microwave and sections were incubated with 3% H_2_O_2_ deionized water to block endogenous peroxidase activity. The sections were blocked with goat serum and incubated with primary antibodies (Wnt4, 1 : 50; p-GSK3*β* (S9), 1 : 200; *β*-catenin, 1 : 100; ILK, 1 : 50; TGF-*β*_1_, 1 : 100) overnight at 4°C. This was followed by biotinylated goat secondary antibody and streptavidin-HRP. Staining was developed with diaminobenzidine (DAB) and then sections were counterstained with haematoxylin, mounted with gummy neutral balsam, and imaged under BX63 + DP72 optical microscope (Olympus Co. Ltd., Tokyo, Japan). Positive results are the fact that the target protein were stained as tan or brown. All immunohistochemical results were repeated at least three times, and the representative images were presented.

### 2.7. Renal Morphologic Analysis

Renal histological analysis was performed on FAA-fixed and paraffin-embedded kidney sections (thickness 2 *μ*m) stained with Hematein Eosin (HE) and periodic acid-Schiff (PAS). Morphology of kidney tissue was observed by light microscopy (Olympus Co. Ltd., Tokyo, Japan). Cortical tissue was fixed using 4% glutaraldehyde and 1% osmium tetroxide, dehydrated by acetone and infiltrated using the mixture of acetone and epoxy resin for 15 min. After double electronic staining by uranium acetate and lead citrate, the ultrastructures of glomeruli were detected by Hitachi H-7650 transmission electron microscopy (Hitachi Ltd., Tokyo, Japan).

### 2.8. Statistical Analysis

All data were expressed as the mean±standard deviation (SD). Statistical analysis was performed using Statistics Product and Service Solutions 17.0 software (SPSS v.17.0 for windows; SPSS Inc., Chicago, IL, USA). One-way ANOVA was used for intergroup comparisons and SNK-*q* test was for the evaluation of differences between two groups. The significance level was defined as *P* < 0.05.

## 3. Results

### 3.1. Important Adverse Events in Each Experimental Group

The rats in C group were normal and there was no death. During the intervention period, 3 rats in M group and 2 rats in HTH group died and 1 rat blood glucose was lower than 16.7 mmol/L, respectively, in the two groups, which was eliminated. In the later stage of intervention, 2 rats appeared to have abnormal symptoms, such as, fester of the skin and the paws and slowness of reaction in M group.

### 3.2. Effects of HTH on the Biochemical Parameters of Rats

**Figure 1:**
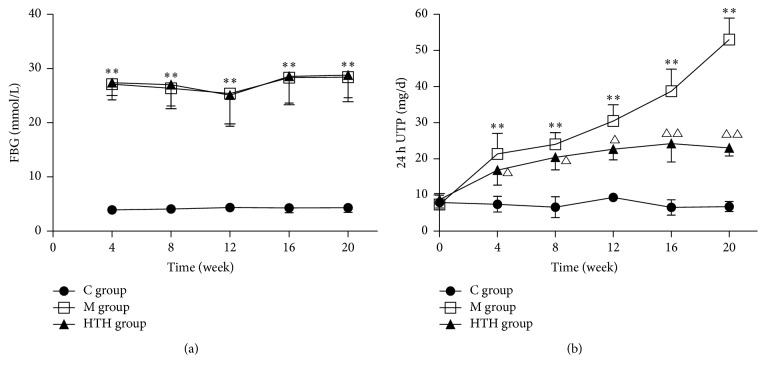
FBG and 24 h UTP changes of tendency in different groups. The changes of tendency in rats' FBG every 4 weeks. Compared with C group, ^*∗∗*^*P* < 0.01 (a). The changes of tendency in rats' 24 h UTP (24 h urine total protein) every 4 weeks. Compared with C group, ^*∗∗*^*P* < 0.01. Compared with M group, ^△^*P* < 0.05, ^△△^*P* < 0.01 (b).

After being fed for 20 weeks, rats were sacrificed and the biochemical and physical characteristics of animals were shown in Figure 1 and Table 3. Body weight (BW) was decreased and serum creatinine (Scr) and blood urea nitrogen (BUN) were significantly increased in M group rats compared with C group rats (*P* < 0.05). As shown in Table 3, HTH group rats attenuated the increases in BUN and the decreases in BW compared with M group rats (*P* < 0.05).

In the study, we measured rats' FBG every 4 weeks. As illustrated in Figure 1(a) FBG in C group rats were normal, while M group rats and HTH treatment group rats showed increase in FBG (*P* < 0.05), which showed intragastric administration of HTH and had no effect on FBG (*P* > 0.05). From the comparison of 24 h UTP every 4 weeks in each group, we known that 24 h urinary protein loss increased with the extension of DN and HTH could reduce the urinary protein leakage in different periods of DN (Figure 1(b)).

### 3.3. Effects of HTH on the Wnt/*β*-Catenin Signaling Pathway of Rats

**Figure 2:**
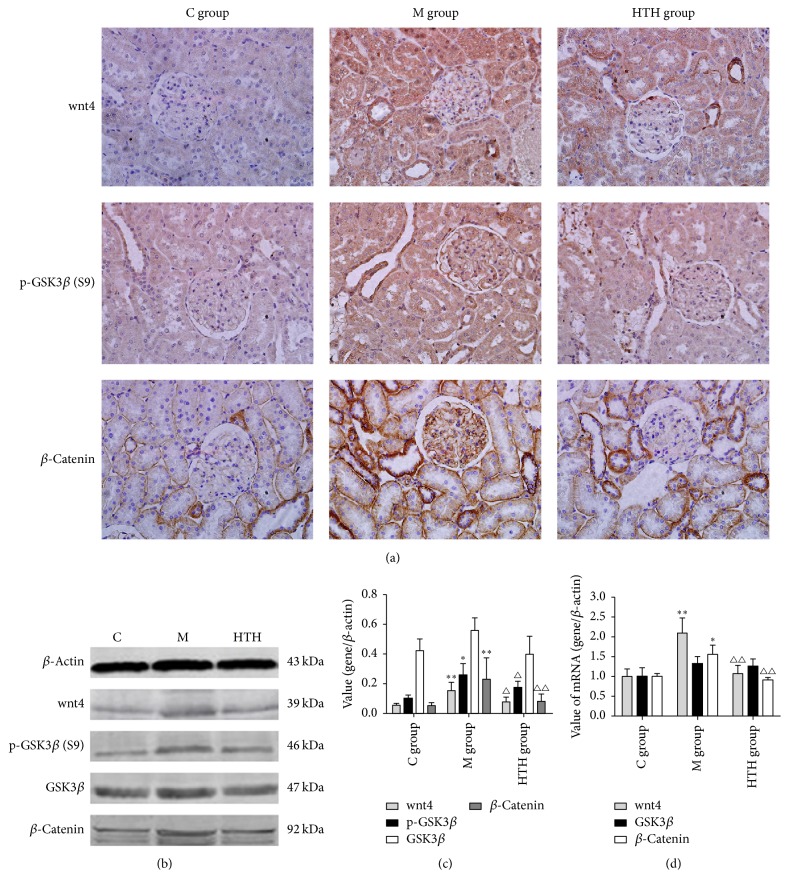
Effects of HTH on the Wnt/*β*-catenin signaling pathway of rats. Immunohistochemical staining for Wnt4, phospho-GSK3*β* (S9), and *β*-catenin in renal cortex of normal control rat group (C group), diabetic model rat group (M group), and Huayu Tongluo herbs treatment rat group (HTH group), exposed to DM and then administration of Chinese medicine of stasis removing and collaterals dredging herbal granule suspension intragastrically (a). Protein levels of Wnt4, phospho-GSK3*β* (S9), GSK3*β*, and *β*-catenin were determined by Western blot and quantified by densitometry in renal cortex of three groups ((b), (c)). Real-time PCR of Wnt4, GSK3*β*, and *β*-catenin mRNA with an internal control of *β*-actin in three groups (d). Values are expressed as means ± SD. Compared with C group, ^*∗*^*P* < 0.05, ^*∗∗*^*P* < 0.01; compared with M group, ^△^*P* < 0.05, ^△△^*P* < 0.01.

To explore the effect of HTH on the expression of Wnt/*β*-catenin signaling pathway in DN rats, we employed a rat model as previously reported to induce DN. The Western blot and PCR results illustrated that, compared with C group, the mRNA and protein expressions of Wnt4 and *β*-catenin in kidney cortex in M group rats were significantly upregulated (*P* < 0.01), and the level of p-GSK3*β* (S9) protein was significantly increased in M group (*P* < 0.05). Meanwhile, HTH significantly decreased the expression of Wnt4, p-GSK3*β* (S9), and *β*-catenin in diabetic rats (*P* < 0.05). The expression of GSK3*β* in each group had no change (*P* > 0.05). In line with Western blot and PCR results, immunohistochemistry also confirmed that Wnt/*β*-catenin signaling pathway overexpression in kidney of DN rats and HTH could reduce the overexpression of aforementioned proteins in Wnt/*β*-catenin signaling pathway (*P* < 0.05) (Figure 2).

### 3.4. Effects of HTH on the Changes in Histopathology of Kidney

**Figure 3:**
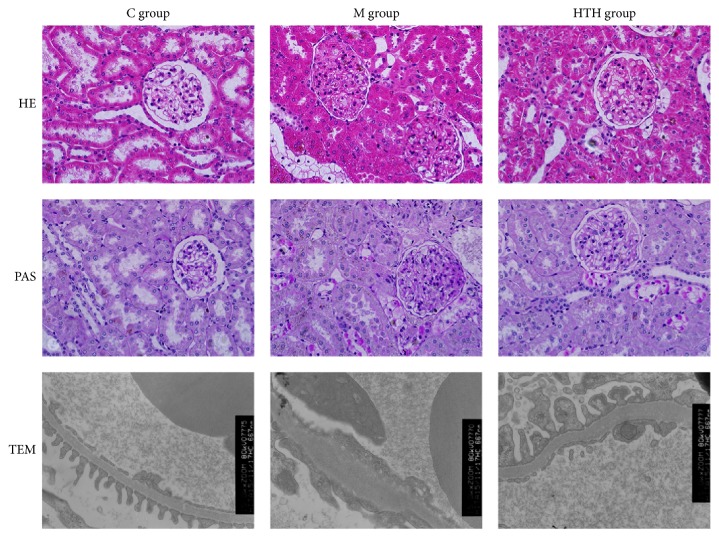
Effects of HTH on the Changes in Histopathology of kidney. Histology of kidney tissue under the light microscope from rat of normal control group (C group), diabetic model group (M group), and Huayu Tongluo herbs treatment group (HTH group) (HE staining ×400, PAS staining ×400). Ultrastructural changes in glomeruli under the electron microscope from rat of the three groups (×15000).

To examine the pathological observation of renal tissue in DN rats, we used light microscope and electron microscope to observe. Light microscope results: the kidney structure was clear and normal in C group. Changes in ultrastructures of glomeruli in the M group showed that the average glomerular diameter was enlarged, glomerular basement membrane (GBM) was thickened, and mesangial matrix was increased. The pathological changes were reduced to a certain extent in the HTH group. Electron microscope results: GBM in C group was regular without fusion in foot processes. In M group, GBM was diffusely thickened, and foot processes showed extensive fusion. By contrast, ultrastructural changes of glomeruli in HTH group were improved with GBM partly thickening and segmental fusion in foot processes. The above pathological changes indicated that the DN model was successful, and HTH could relieve renal pathological damage (Figure 3).

### 3.5. Effects of HTH on E-Cadherin, ILK, and TGF-*β*_1_ of Rats

**Figure 4:**
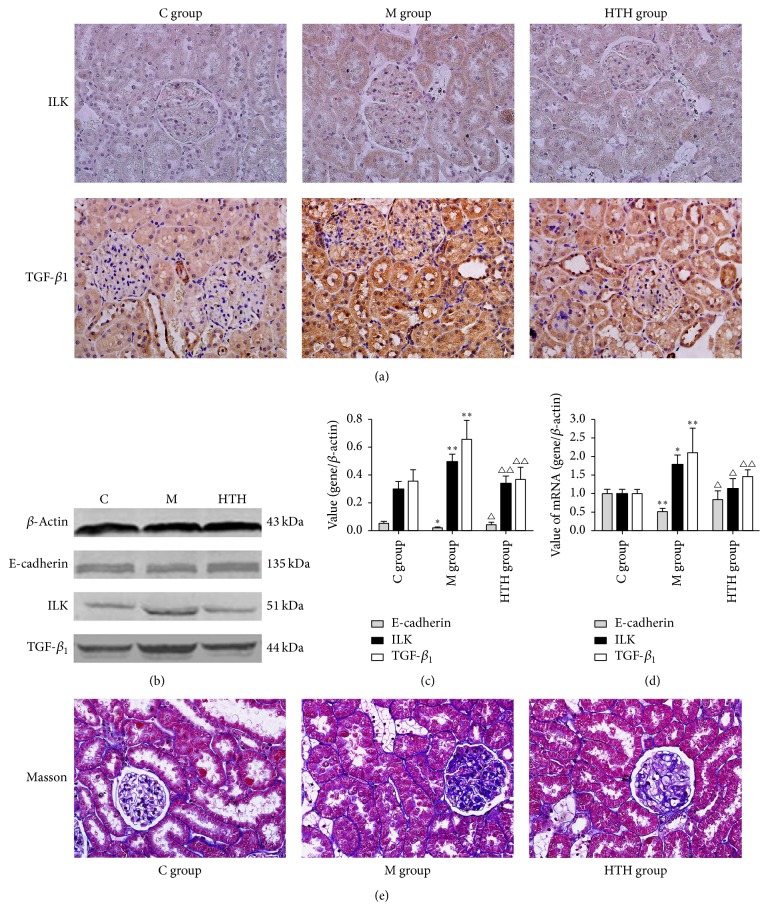
Effects of HTH on E-cadherin, ILK, and TGF-*β*_1_ of rats. Immunohistochemical staining for ILK and TGF-*β*_1_ in the kidneys of rat of normal control rat group (C group), diabetic model rat group (M group), and Huayu Tongluo herbs treatment rat group (HTH group), exposed to DM and then administration of Chinese medicine of stasis removing and collaterals dredging herbal granule suspension intragastrically (a). Western blot detection and statistical analyses for renal E-cadherin, ILK, and TGF-*β*_1_ in kidneys of rat of three groups ((b), (c)). Quantification of E-cadherin, ILK, and TGF-*β*_1_ mRNA expression levels in different groups by Real-time PCR (d). Masson trichrome staining revealed extracellular matrix deposition in the kidneys of rat of three groups (stained in blue) (e). Values are expressed as means ± SD. Compared with C group, ^*∗*^*P* < 0.05, ^*∗∗*^*P* < 0.01; compared with M group, ^△^*P* < 0.05, ^△△^*P* < 0.01.

It can be seen in Figure 4 that the expressions of ILK and TGF-*β*_1_ in kidneys of DN rat stained as brown yellow granules were decreased notably with HTH. Also, the results of Western blot and PCR revealed that the expressions of ILK and TGF-*β*_1_ were significantly upregulated in M group in comparison with the C group (*P* < 0.05). Again, renal E-cadherin mRNA and protein were lowered in M group rats compared with C group rats, and that expression in HTH group rats increased compared with M group rats (*P* < 0.05). Furthermore, Masson staining showed ECM accumulation located in the tubulointerstitium and part of glomeruli of DN rat was alleviated with HTH.

## 4. Discussion

In Chinese medicine, long duration of diabetes lead to collateral stasis in kidney, causing the glomerular capillary group structure and function disorder, which result in leakage of urine protein. Clinical reports and studies have confirmed that almost all the DN patients had different degree manifestation of blood stasis syndrome, throughout the course of the disease [14–16]. So the pathogenesis of DN focused on stagnant blood obstructing collaterals. In this point of view, we organized Huayu Tongluo herbs as basic medicine to treat DN. HTH has exhibited its renal protective effect in the treatment of DN patients; it could alleviate clinical symptoms of patients, reduce urinary protein leakage, and improve the quality of life [17]. Among the herbs,* Rhizoma Chuanxiong* and* Radix Salviae Miltiorrhizae* may promote the circulation of blood;* Pheretima*,* Hirudo*, and* Scorpio* are able to dredge collaterals. HTH intend to eliminate collateral stasis in kidney, improve renal microcirculation, and delay the renal fibrosis. Previous studies have confirmed that HTH can inhibit the activation of RAS system in kidneys of DN rats, reduce the loss of podocyte slit diaphragm proteins, maintain the integrity of the filtration barrier, and reduce the leakage of proteinuria [11, 12, 18, 19]. This study aimed to discuss the mechanisms of HTH reducing urinary protein and protecting podocyte.

Proteinuria not only is the main clinical manifestation of DN, but also is closely related to the severity and progression of DN. The continuous activation of the Wnt signaling pathway in renal podocytes was commonly involved in the development of proteinuria and glomerulosclerosis [5, 20]. Many recent studies have shown that the Wnt/*β*-catenin signaling pathway was closely related to diabetic nephropathy [21, 22]. And it has been confirmed in recent years that high glucose can activate this pathway [23]. The activation of Wnt signal and the expression of nuclear translocation of *β*-catenin were involved in the development of podocyte injury and proteinuria [24]. A study's results indicated that Wnt/*β*-catenin signaling in podocytes played a critical role in integrating cell adhesion, motility, cell death, and differentiation [25]. In the signaling pathway, Wnt4 is a secreted glycoprotein that is critical for genitourinary development but is found only in the most distal collecting duct epithelium in the normal murine adult kidney [26]. Wnt4 protein plays an important role in the formation of renal tubules, renal fibrosis induced glomerulosclerosis, and proteinuria [27]. In the pathway of Wnt/*β*-catenin, *β*-catenin, a cytosolic protein that is degraded by the ubiquitin proteasome system by glycogen synthase kinase 3 beta (Gsk3*β*) phosphorylation, is the key factor of the canonical Wnt signaling pathway. *β*-catenin was involved in the process of cell proliferation, differentiation, and apoptosis and had the function of mediating intercellular adhesion and gene expression [28]. Animal experiments showed that DN is accompanied by the activation of Wnt signaling pathway, leading to increased expression of downstream factor *β*-catenin, which regulated the transcription of downstream target genes, thereby affecting cell differentiation, proliferation, apoptosis, migration, and other functions [29]. Inappropriate activation of the Wnt/*β*-catenin pathway has been indicated in podocyte dysfunction and injury and shown to contribute to the development and progression of nephropathy [30]. Experiments showed that the activation of this pathway may be involved in ADR-induced podocyte foot process effacement, disruption of the slit diaphragm, and consequent albuminuria [31]. And another study data revealed that careful contemplation is required when targeting Wnt/*β*-catenin pathway to treat proteinuric kidney diseases associated with impaired CD2AP [32]. Therefore, Wnt/*β*-catenin pathway is important in modulating the slit diaphragm proteins of the filtration barrier. In this experiment, the protein and mRNA levels of Wnt4, p-GSK3*β*, and *β*-catenin were markedly increased in M group and decreased by HTH treatment group. But protein and mRNA level of GSK3*β* were not changed. Our study showed that the high expression of Wnt/*β*-catenin pathway was involved in the formation of a large number of proteinuria in diabetic nephropathy rats and HTH could inhibit the high expression of the pathway.

Renal interstitial fibrosis is an inevitable outcome of all causes of progressive chronic kidney disease (CKD), including DN [33]. The characteristic pathological changes of renal interstitial fibrosis include renal tubular atrophy, interstitial infiltration of inflammatory cells, fibroblasts accumulation, and extracellular matrix (ECM) deposition. In the study, Masson staining presented the fact that marked ECM deposition (stained in blue) was found in tubulointerstitium and part of glomeruli of DN rat in contrast to control rat. The results showed that HTH can significantly reduce the accumulation of ECM. The hallmark of progressive DN is ECM production leading to renal fibrosis, and Transforming Growth Factor-beta (TGF-*β*) is generally considered as a central mediator of fibrotic diseases [34, 35]. Integrin-linked kinase (ILK) is an ankyrin-repeat containing serine/threonine protein kinase that plays an important role in the regulation of cell adhesion, survival, proliferation, and ECM deposition [36]. It has been proved to be a key mediator of TGF-*β* induced fibrosis. And ILK is a critical mediator for tubular EMT and likely plays a crucial role in the pathogenesis of chronic renal fibrosis [37]. Furthermore, ILK is independently identified as a key mediator of podocyte dysfunction, proteinuria, podocyte cell matrix interaction, and slit membrane gene expression in many forms of proteinuric kidney diseases [38, 39]. In addition, the activity of ILK enables directly phosphorylating important downstream effector proteins, such as GSK3*β* [40]. ILK activation has been shown to decrease matrix adhesion concomitant with a downregulation of E-cadherin [41]. Meanwhile, reduction or loss of E-cadherin expression is one of the well-established hallmarks of epithelial-mesenchymal transitions (EMT) [42]. Experiments in vivo and in vitro have found that high glucose could lead to EMT in podocytes. In our study, the protein and mRNA level of E-cadherin were markedly decreased in M group and increased by HTH. TGF-*β*1 and ILK, as important fibrogenic factor, were upregulated in the DN kidneys in experimental animal models, and expression levels were significantly decreased in HTH group.

But our research still had some limitations. Firstly, there was the pathogenesis of DN; in addition to the “obstruction of collaterals by blood stasis,” some scholars believe that the spleen and kidney deficiency and damp heat and phlegm are involved. There are many kinds of treatment methods for DN, and no matter which period of DN, the treatment of removing blood stasis and dredging collaterals is the basic treatment. Secondly, the present study only focused on the Wnt/*β*-catenin pathway signaling pathway. Activation of other signaling pathways may also be associated with increasing DN proteinuria, such as PI3K/AKT and Notch signaling pathways. Thirdly, in this study, DN rat model was established by high-fat diet and low dose of STZ. Due to various factors, such as diet or drugs, the model may still have differences with the occurrence of clinical diseases. Finally, the systematic error (reagent batch variance, instrument errors, etc.) may lead to the imprecision of results. Therefore, this research needs to be in-depth study and further clinical application is needed.

## 5. Conclusions

In summary, our results indicated that, in diabetic nephropathy rats, Huayu Tongluo herbs could relieve both the structural damage and functional changes significantly. And high glucose activated the expression of Wnt/*β*-catenin pathway; renal fibrosis existed in diabetic nephropathy rats. Huayu Tongluo herbs may obviously inhibit the high expression of Wnt/*β*-catenin pathway, which might be associated with relieving podocyte injury and renal fibrosis. Also, our data provided a potential target for prevention or treatment of proteinuria resulting from diabetes nephropathy.

## Figures and Tables

**Table 1:** Format of Chinese herbal granules applied in the study.

Granule name	Lot number	Packing size (g/package)	Equivalent to crude drug (g)
Danshen *(Radix Salviae Miltiorrhizae)*	501306T	1.8	10
Chuanxiong *(Rhizoma Chuanxiong)*	412272T	1.3	6
Dilong *(Pheretima Aspergillum)*	501153T	1.0	10
Shuizhi *(Hirudo)*	408245T	1.5	3
Quanxie *(Scorpio)*	412299T	1.0	3

**Table 2:** Primers for rat genes used in real-time PCR.

Gene	Forward primer (5′→3′)	Reverse primers (5′→3′)	Length of product (bp)
*β*-Actin	ACCCGCGAGTACAACCTTCT	TTCAGGGTCAGGATGCCTCT	266 bp
Wnt4	AGCCCACAGGGTTTCCA	GCTCGCCAGCATGTCTTT	233 bp
GSK-3*β*	GTCCGATTGCGGTATT	GTGTCTGGCGACTCTGTA	108 bp
*β*-Catenin	AACGGCTTTCGGTTGAGCTG	TGGCGATATCCAAGGGCTTC	147 bp
E-Cadherin	TGCTCCTACTGTTTCTACG	CTTCTCCACCTCCCTCTT	111 bp
ILK	AATGGGACCCTGAACAAA	GAGCCTGGCAAGCACCTA	244 bp
TGF-*β*_1_	ATGGTGGACCGCAACAAC	TGAGCACTGAAGCGAAAGC	329 bp

**Table 3:** Clinical parameters of different groups.

Parameters	C group	M group	HTH group
Body weight (g)	554.50 ± 43.78	272.22 ± 32.67^*∗∗*^	322.21 ± 35.02^△△^
Blood urea nitrogen (mmol/L)	6.89 ± 0.76	17.76 ± 1.54^*∗∗*^	12.91 ± 3.84^△△^
Serum creatinine (*μ*mol/L)	24.08 ± 4.31	40.99 ± 9.52^*∗*^	32.43 ± 7.87

*Notes*. Compared with C group, ^*∗*^*P* < 0.05, ^*∗∗*^*P* < 0.01; compared with M group, ^△△^*P* < 0.01.
